# Cone Identification in Choroideremia: Repeatability, Reliability, and Automation Through Use of a Convolutional Neural Network

**DOI:** 10.1167/tvst.9.2.40

**Published:** 2020-07-16

**Authors:** Jessica I. W. Morgan, Min Chen, Andrew M. Huang, Yu You Jiang, Robert F. Cooper

**Affiliations:** 1 Scheie Eye Institute, Department of Ophthalmology, University of Pennsylvania, Philadelphia, PA, USA; 2 Center for Advanced Retinal and Ocular Therapeutics, University of Pennsylvania, Philadelphia, PA, USA; 3 Department of Radiology, University of Pennsylvania, Philadelphia, PA, USA; 4 Department of Psychology, University of Pennsylvania, Philadelphia, PA, USA; 5 Currently at the Joint Department of Biomedical Engineering, Marquette University and Medical College of Wisconsin and the Department of Ophthalmology, Medical College of Wisconsin, Milwaukee, WI, USA

**Keywords:** adaptive optics, choroideremia, convolutional neural network, cones

## Abstract

**Purpose:**

Adaptive optics imaging has enabled the visualization of photoreceptors both in health and disease. However, there remains a need for automated accurate cone photoreceptor identification in images of disease. Here, we apply an open-source convolutional neural network (CNN) to automatically identify cones in images of choroideremia (CHM). We further compare the results to the repeatability and reliability of manual cone identifications in CHM.

**Methods:**

We used split-detection adaptive optics scanning laser ophthalmoscopy to image the inner segment cone mosaic of 17 patients with CHM. Cones were manually identified twice by one experienced grader and once by two additional experienced graders in 204 regions of interest (ROIs). An open-source CNN either pre-trained on normal images or trained on CHM images automatically identified cones in the ROIs. True and false positive rates and Dice's coefficient were used to determine the agreement in cone locations between data sets. Interclass correlation coefficient was used to assess agreement in bound cone density.

**Results:**

Intra- and intergrader agreement for cone density is high in CHM. CNN performance increased when it was trained on CHM images in comparison to normal, but had lower agreement than manual grading.

**Conclusions:**

Manual cone identifications and cone density measurements are repeatable and reliable for images of CHM. CNNs show promise for automated cone selections, although additional improvements are needed to equal the accuracy of manual measurements.

**Translational Relevance:**

These results are important for designing and interpreting longitudinal studies of cone mosaic metrics in disease progression or treatment intervention in CHM.

## Introduction

Adaptive optics (AOs) ophthalmoscopy, including adaptive optics scanning laser ophthalmoscopy (AOSLO),^1^ has enabled high-resolution observation of the living human retina both in health and disease.^2^^,^^3^ Main advantages of AO ophthalmoscopy include the ability to observe single cells in vivo and to track those same cells over time. Indeed, AO ophthalmoscopy has been used to describe the degeneration of cone photoreceptor structure using metrics, such as cone density or spacing, in numerous inherited retinal diseases, including retinitis pigmentosa,^4^^–^^7^ Stargardt's,^8^^–^^10^ achromatopsia,^11^^–^^14^ and choroideremia (CHM),^15^^–^^17^ among others.^2^^,^^3^ In addition, investigators have demonstrated longitudinal imaging of the same photoreceptors over time.^18^^–^^20^

Despite these advantages, challenges remain for translating AOSLO imaging into large-scale clinical studies to follow disease progression. Most AO studies to date have been performed through cross-sectional analysis and include only a relatively small number of patients.^2^ As studies transition from small cross-sectional studies to larger longitudinal studies, investigators need to know the reliability with which they can quantify mosaic metrics and will need to obtain those measurements within a reasonably quick time period. The present study considers these issues within the context of one inherited retinal degeneration, choroideremia (CHM).

CHM is an X-linked inherited retinal degeneration caused by mutations in the *CHM* gene leading to nonfunctional Rab escort protein-1.^21^^,^^22^ Patients present with nyctalopia and constricted visual fields, leading to tunnel vision and ultimately blindness.^23^ Clinical retinal imaging of CHM has shown central islands of retained retinal structure with sharp borders demarcating a narrow transition zone between retained and atrophic retinal areas showing loss of the photoreceptors, retinal pigment epithelium, and choroid.^24^ Cross-sectional studies using AOSLO imaging have revealed patients with CHM have a contiguous photoreceptor mosaic within their central islands, with some regions showing normal or near normal cone density, whereas others show reduced cone density.^15^^–^^17^ Functional sensitivity testing with AO microperimetry has revealed close correspondence between retained retinal function and structure, with sharp losses in function being co-located with the sharp structural transitions between intact and atrophic retina.^25^

Previous AOSLO cross-sectional studies investigating CHM used manual identification of cone locations to quantify the cone photoreceptor phenotype.^15^^,^^16^ Although manual cone identification is considered the gold-standard for assessing cone mosaic metrics,^26^ the intragrader repeatability and intergrader reliability for cone density measurement in CHM remains unknown. For studies that aim to show true retinal change through longitudinal analysis, this information must be understood.

In addition, manual analysis of cone density requires a large amount of grader effort. As a result, there remains a trade-off between including more images/time points/patients in a study and completing the study within a short timeframe. Using fully automated methods to identify cones could offer a substantial time-saving advantage. Recent advances have demonstrated that a convolutional neural network (CNN) may be trained to identify cone locations within normal AOSLO images and shows good agreement with manual cone identifications.^27^ Retraining the network using multimodal images from patients with achromatopsia has resulted in automated cone identifications in achromatopsia images with good agreement to manual identifications.^28^ In addition, a multidimensional recurrent neural network has been shown to yield automatic cone identifications in Stargardt disease in good agreement with manual identifications.^29^ However, it remains to be determined to what extent these techniques can be applied to patients with other retinal diseases as different diseases present with varying phenotypes in AOSLO images.^2^

In the present study, we address the issues described above for translating quantifications of cone metrics for CHM into ones that can be readily applied to longitudinal clinical trials. We asked: to what extent are cone identifications and cone density measurements repeatable and reliable in CHM? To answer this question, we investigated intragrader repeatability and intergrader reliability for identifying cone locations and quantifying cone density using non-confocal split-detection AOSLO images showing the inner segment cone mosaic in patients with CHM. We then asked, to what extent do automatic cone identifications from an open-source CNN-based algorithm agree with manual cone identifications in CHM? To answer this, we used an open-source CNN pretrained on normal images or retrained with CHM images to automatically identify cones, and compared the CNN automated cone identifications to the manual identifications and the results found for intragrader and intergrader agreement.

## Methods

This research followed the Declaration of Helsinki and was approved by the Institutional Review Board at the University of Pennsylvania. Following an explanation of the study, all patients gave informed consent or assent with parental permission, and were voluntarily enrolled in the study.

Seventeen eyes from 17 patients with CHM were included in the study. Axial lengths of each eye were measured using an IOL Master examination (Carl Zeiss Meditec, Dublin, CA). AOSLO images were scaled proportionally by axial length as has been done previously.^30^^,^^31^

The AOSLO used in this study has been previously described.^32^^,^^33^ Patients were aligned to the imaging system using a dental impression. Wavefront sensing was performed with an 848 Δ26 nm superluminescent diode (Superlum, Cork, Ireland). Aberration correction was performed using a 97-actuator deformable mirror (Alpao SAS, St. Martin, France). Multimodal imaging was performed with a 795 Δ15.3 nm superluminescent diode (Superlum) and three photomultiplier tubes (Hamamatsu Corporation, Naka-ku, Japan) configured for confocal and nonconfocal split-detection reflectance imaging.

Patients with CHM were instructed to fixate with the imaging eye at a target. AOSLO image sequences were acquired over the central 3 x 3 degrees surrounding fixation and along all four meridians. Image sequences were desinusoided, a reference frame was automatically chosen using a custom MatLab (The MathWorks Inc., Natick, MA) algorithm based on the method published by Salmon et al.,^34^ and 50 frames were registered using custom software that removes intra-frame distortions caused by eye motion.^35^ Registered images were then averaged together and the averaged images were “dedistorted” using a custom MatLab algorithm based on the method published by Bedggood and Metha^36^ to remove distortions caused by eye motion from the reference frame. These images were then automatically montaged using a custom algorithm as previously described.^37^ Regions of interest (ROIs) along the retinal meridians showing the cone photoreceptor inner segment mosaic in the nonconfocal, split-detection imaging modality were manually selected from the montages. A total of 204 ROIs were cropped from the 17 CHM montages (range: 5–16 ROIs per montage). ROI locations ranged from 135 to 2210 µm from fixation, with an average of 468 ± 316 µm (mean ± SD). ROIs were square with 70 ± 22 µm sides (mean ± SD). These 204 ROIs were then used to assess intra-observer repeatability and inter-observer reliability for manual cone identifications and the quality of automated cone identifications through use of an open-source CNN.^27^ Each experiment is described in detail below.

### Intra-Observer Repeatability

An experienced grader (grader 1, J.I.W.M.) manually identified cones in the split-detection images of all 204 ROIs using custom software (MOSAIC; Translational Imaging Innovations). Throughout the remainder of the text, we will refer to these cone identifications as grader 1A. Each ROI was presented in a randomized order and the grader was masked to the subject ID and retinal location of the image. The grader was able to adjust contrast, brightness, and magnification of the ROI image while manually identifying cones by clicking on the center of each cell to record the cone location. Six of the patients’ images also had the confocal image available for viewing, although the grader was instructed to use the split detection image as the primary source for cone identifications.

Grader 1 then regraded the ROIs by manually identifying all the cones in the split-detection image a second time using the same custom software (MOSAIC; Translational Imaging Innovations); we will refer to this set of cone identifications as grader 1B. The gradings were separated by a minimum of 6 months. The grader was again masked to subject ID and retinal location and the images were presented in a randomized order.

We then compared the cone identifications made by grader 1A to grader 1B. Using grader 1A as the ground truth, we calculated the true positives, false positives, and false negatives in grader 1B by comparing the list of cone coordinates. To be considered a true positive, a cone would need to be marked in both sets of grades. To find cones marked in both sets of grades, we first combined the coordinate lists of both grading into one master list of coordinates. We then found the nearest coordinate for each selection in the master list and used the mean nearest coordinate distance plus two times the SD of the nearest coordinate distance as the threshold distance for determining whether a marking from the second set was considered the same cone as a cone identified in the first set.^31^ Cones that fell outside of this distance were considered separate cones. If more than one cone from the second grading fell within the threshold distance from a cone in the first grading, the closer of the two cones was considered the match. From this grouping between cone identifications, we determined the number of true positives (*N_TP_*, cones that were identified at the same location for both sets of grades), false positives (*N_FP_*, cones that were identified in grader 1B but not grader 1A), and false negatives (*N_FN_*, cones that were identified in grader 1A but not grader 1B). The total number of cone identifications made can be expressed as:

(1) $$N_{comparison\_set}=N_{TP}\;+N_{FP}$$

(2) $$N_{ground\_truth\_set}=N_{TP}\;+N_{FN}$$

We then measured the true positive rate, the false positive rate, and Dice's coefficient^38^ (a metric for assessing similarity) between the two sets of cone identifications, given by the following equations:

(3) $$true\;positive\;rate\;=N_{TP}\;/N_{ground\_truth\_set}$$

(4) $$false\;positive\;rate=N_{FP}\;/N_{comparison\_set}$$

(5) $$\begin{array}{ccc} Dice{}^{'}s\;coefficient & = & 2N_{TP}\;/(N_{ground\_truth\_set}\\ & & +N_{comparison\_set}) \end{array}$$

For the intragrader repeatability analysis, *N*_*Grader* 1*A*_ was used as $N_{ground\_truth\_set}\;$and *N*_*Grader* 1*B*_ was used as $N_{comparison\_set}$.

In addition to assessing the precision of repeated cone identifications, we also compared the bound cone densities calculated from each grading. Bound cone density reduces boundary effects by excluding border cones from the analysis. To identify border cones, a Voronoi analysis was performed for each ROI with each set of cone identifications and cones that did not have a complete Voronoi area within the image were excluded from density calculations. Bound cone density was then calculated as the number of cones with complete Voronoi areas within the image divided by the sum of their Voronoi areas, as previously described.^39^ The two calculated bound cone densities for the ROI were then compared using Bland-Altman analysis.^40^

### Interobserver Reliability

Two additional experienced graders (grader 2, R.F.C. and grader 3, G.K.V.) also manually identified cones in the same 204 ROIs using the same custom software (MOSAIC; Translational Imaging Innovations). Again, the graders could adjust image brightness, contrast, and magnification, were masked to subject ID and retinal location, and images were presented in a randomized order. Again, bound cone density was calculated for each ROI for each grader.

We then compared these graders’ results with the results from grader 1A, described above. Using methods previously described,^31^ we combined the cone selections from all graders into a master coordinate list and clustered cone locations for each ROI across all graders. From this master list, we grouped cone locations that were located within the mean nearest coordinate distance plus two SDs of each other. Only one cone identification per grader was allowed in a cluster. We then assessed the similarity between graders’ cone identifications using pairwise comparisons and rotating the grader who was considered ground truth. (For example, first considering grader 1A as ground truth, and comparing grader 2 to 1A and grader 3 to 1A. Then, considering grader 2 as ground truth, and comparing grader 1A to 2 and grader 3 to 2, etc.) We then found the true positives (*N_TP_*, ground truth grader and comparison grader both identified a cone), false positives (*N_FP_*, comparison grader identified a cone but ground truth grader did not), and false negatives (*N_FN_*, ground truth grader identified a cone but the comparison grader did not). As before, we found the true positive rate, false positive rate, and Dice's coefficient between graders using Equations 3 to 5 above.

We compared the rates between graders and the intra-observer rates found from grader 1 using a repeated measures 1-way analysis of variance (ANOVA) with significance assessed at *P* < 0.05. We then performed post hoc *t*-tests using pairwise comparisons, including a Bonferroni correction to adjust for multiple comparisons. In addition, we compared the bound cone densities calculated from each grader's cone identifications using interclass correlation coefficient (ICC) with 95% confidence intervals (CIs).

### Automated Cone Identification Using an Open-Source Convolutional Neural Network

We used the open-source split-detection trained CNN published in Cunefare et al.^27^ to automatically identify cones in the 204 nonconfocal split-detection CHM images. This CNN was pretrained on nonconfocal split-detection images located from 0.5 to 2.8 mm from fixation in normal retinas. We then compared the output of the pretrained CNN (termed normal-CNN) to grader 1A, using grader 1A as ground truth. We identified true positives (*N_TP_*, both grader 1A and normal-CNN), false positives (*N_FP_*, normal-CNN identified a cone but grader 1A did not), and false negatives (*N_FN_*, grader 1A identified a cone but normal-CNN did not). We then measured the true positive rate, the false positive rate, and Dice's coefficient using Equations 3 to 5 where *N*_*Grader* 1*A*_ was used as $N_{ground\_truth\_set}\;$and *N*_*normal* − *CNN*_ was used as $N_{comparison\_set}$.

We then retrained the open-source CNN using the 204 CHM split-detection images and grader 1A cone locations. We used a leave-one-subject-out cross-validation approach, as previously published^28^: we trained the network on the images from 16 CHM subjects and used the 17th subject's images as the validation set for that training run. We ran 17 rounds of cross-validation, using each of the 17 subjects’ images as the validation set one time. We then compared the true positive and false positive rate and Dice's coefficient for the cone identifications made by the CHM-trained CNN (termed CHM-1A-CNN) in comparison to grader 1A. For Equations 3 to 5, *N*_*Grader* 1*A*_ was still used as $N_{ground\_truth\_set}\;$but *N*_*CHM* − 1*A* − *CNN*_ was used as $N_{comparison\_set}$. We then used the paired *t*-test to compare true and false positive rates and the Dice coefficient for the normal-CNN and the CHM-1A-CNN, with statistical significance assessed for *P* < 0.05.

Finally, we retrained the CNN with cone identifications made by grader 1B, grader 2, and grader 3. In each case, we ran 17 rounds of training and cross-validation using the leave-one-subject-out approach. These experiments are termed CHM-1B-CNN, CHM-2-CNN, and CHM-3-CNN. We again measured the true and false positive rates and Dice's coefficient for the CNN cone identifications in comparison with the manual identifications used for training the network. We then compared the rates found for CHM-1B-CNN, CHM-2-CNN, and CHM-3-CNN with the rates found for CHM-1A-CNN. In addition, we found the true and false positive rates and Dice's coefficient from the CHM-1A-CNN compared to grader 1B as ground truth and CHM-1B-CNN compared to grader 1A as ground truth. Again, statistical significance was assessed by the repeated measures ANOVA, and post hoc paired *t*-tests corrected for multiple comparisons.

## Results

**Figure 1.**
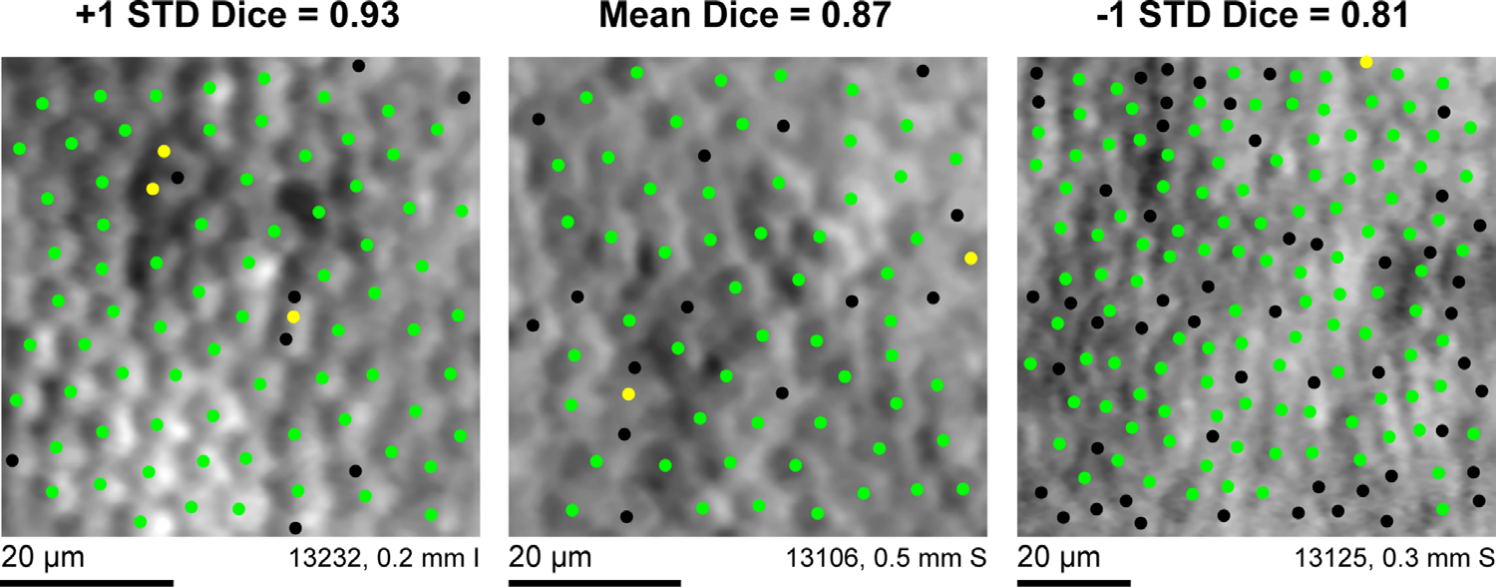
Intragrader repeatability for manual cone selections. Grader 1 exhibited high repeatability for identifying cone locations in the 204 ROIs. *Green dots* show cones that were selected during both the first and second set of manual identifications separated by 6 months by grader 1. *Yellow dots* show cone locations that were selected during the first set of identifications but not the second. *Black dots* show cone locations that were selected during the second set of identifications but not the first. The three images shown are the images corresponding to the mean and the mean ± 1 SD Dice coefficient for the 204 ROIs in the dataset. I = inferior, S = superior.

**Figure 2.**
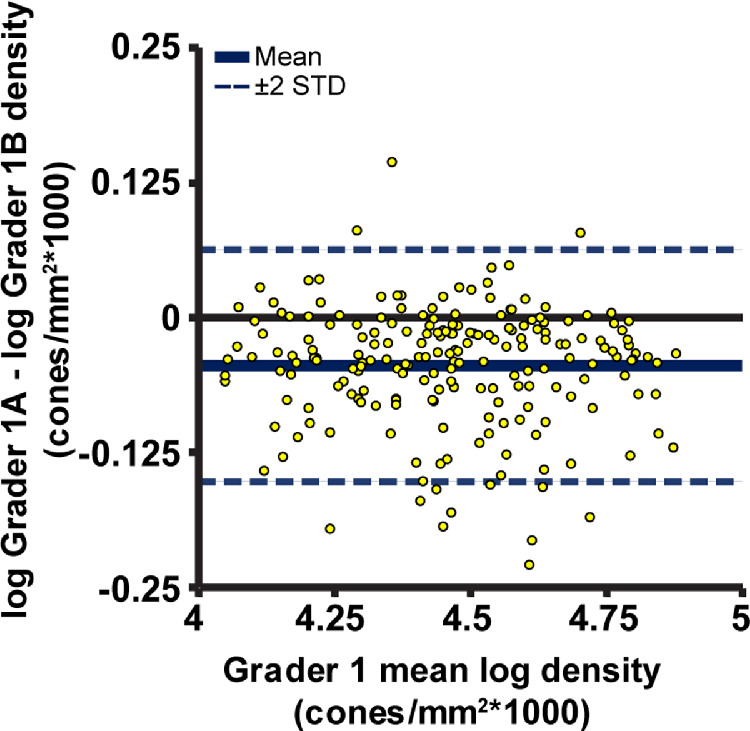
Bland Altman plot for repeat measures of log_10_ bound cone density arising from grader 1's repeated cone selections over the 204 ROIs. The second set of grader 1 cone selection resulted in higher cone densities, on average, with a proportional effect of bound density calculated from the first set of cone selections.

Seventeen eyes of 17 genetically confirmed patients with CHM ages 7 to 43 were included in the study (Table 1). Intragrader agreement was good; grader 1 showed high repeatability when identifying cones within the 204 ROIs, where repeated grades were separated by at least 6 months (Fig. 1). The true positive and false positive rates and Dice coefficient for grader 1's two sets of cone identifications were 0.94 ± 0.05, 0.18 ± 0.09, and 0.87 ± 0.06, respectively (Table 2) when using grader 1A as the ground truth. Grader 1B, on average, resulted in higher bound cone densities than grader 1A, *P* < 0.0001. The differences between the two sets of cone densities failed a test for normality, so the data were log_10_ transformed before Bland Altman analysis was performed. Figure 2 shows grader 1B selections resulted in a higher bound cone density than grader 1A, with a proportional effect; in general, the higher the density for a given image, the greater the difference in density between gradings.

**Table 1.** Patient Demographics

				Axial		Confocal	
				Length	Number of	Images	
Patient #	Study ID	Age	Eye	(mm)	Images	Available	Genetic Mutation
1	13031	14	OD	22.71	16	No	Deletion of exons 3–8
2	13035	22	OS	24.52	13	Yes	c.315_318delTCAG
3	13048	26	OD	23.81	9	No	c.700A>T; p.Lys234Term (K234X)
4	13106	31	OD	24.26	15	No	IVS7-1 G>C c.940-1G>T
5	13122	21	OD	24.46	14	No	c.808C>T; p.Arg270Stop
6	13125	32	OS	23.33	15	Yes	c.1437dupA; p.Glu480ArgfsX12
7	13131	37	OD	24.99	12	Yes	CHM - Met443del2aacAT hemizygous
8	13159	43	OS	25.23	15	Yes	Glu382Stop, GAA>TAA hemizygous
9	13173	28	OD	23.98	14	No	c.757 C>T; p.Arg253Stop(R253X)
10	13183	37	OD	23.02	15	No	c.745delT; p.Ser249LeufsX42
11	13190	22	OD	23.32	7	No	deletion of exons 6, 7, and 8
12	13193	12	OD	24.33	12	No	deletion involving the whole CHM gene
13	13195	37	OD	23.41	16	No	c.820-2A>G
14	13220	8	OD	21.38	8	No	hemizygous c.277A>T p.Lys93Ter(K93X)
15	13226	39	OD	24.06	5	Yes	c.940-2A>T
16	13231	7	OS	22.29	8	Yes	CHM Arg267Stop CGA>TGA hemizygous
17	13232	32	OD	23.96	10	No	CHM Gln380Stop CAA>TAA hemizygous
	Average ± SD	26.35 ± 11.18		23.71 ± 0.98	12 ± 3.5		

**Table 2.** Intra- and Intergrader Agreement in Manual Cone Identifications

	True positive rate	False positive rate	Dice's coefficient
Observer	Mean (SD)	Mean (SD)	Mean (SD)
Grader 1A as “ground truth”			
Grader 1B	0.94 (0.05)	0.18 (0.09)	0.87 (0.06)
Grader 2	0.88 (0.13)	0.16 (0.09)	0.85 (0.09)
Grader 3	0.88 (0.11)	0.23 (0.11)	0.81 (0.08)
Grader 2 as “ground truth”			
Grader 1A	0.84 (0.09)	0.12 (0.13)	0.85 (0.09)
Grader 3	0.86 (0.12)	0.22 (0.15)	0.80 (0.12)
Grader 3 as “ground truth”			
Grader 1A	0.77 (0.11)	0.12 (0.11)	0.81 (0.08)
Grader 2	0.78 (0.15)	0.14 (0.12)	0.80 (0.12)

**Figure 3.**
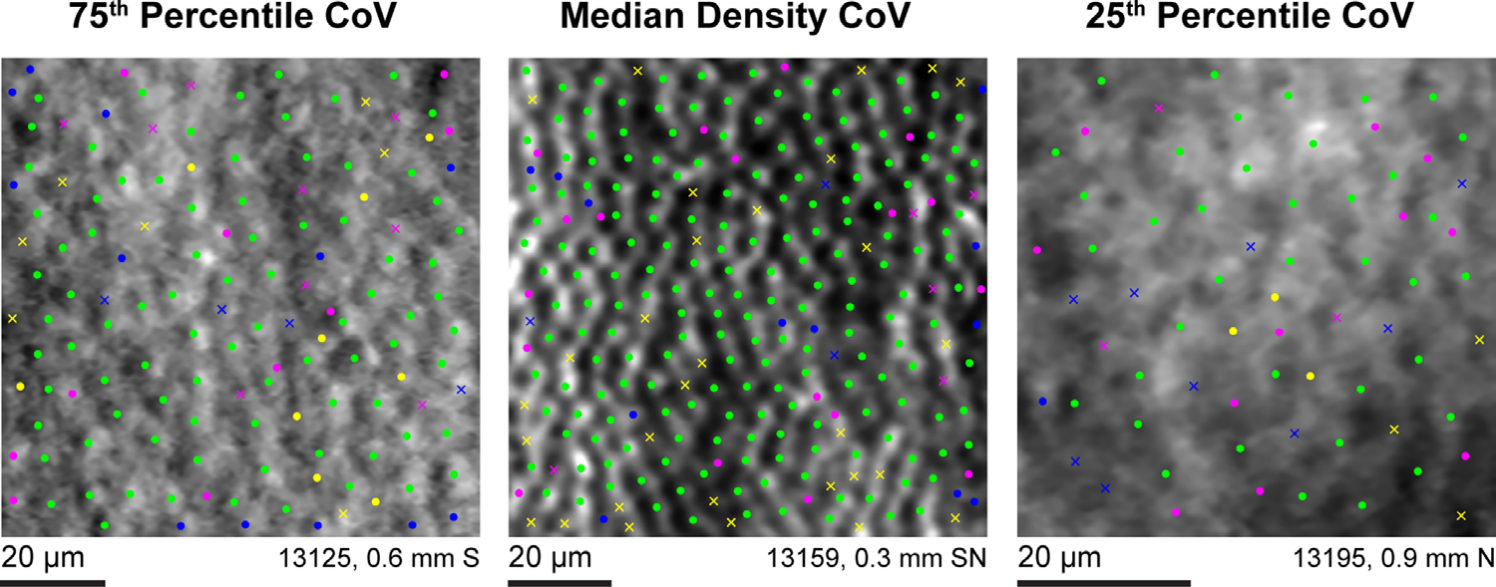
Intergrader reliability for manual cone selections. *Green dots* represent locations where all three graders identified a cone. *Colored dots* represent locations where an individual grader identified a cone. *Colored x's* represent locations where two of the three graders identified a cone, with the color corresponding to the grader who did not identify a cone at that location. Yellow = grader 1A, blue = grader 2, and magenta = grader 3. The three images shown represent the median, 25th, and 27th percentiles for the coefficient of variation (CoV) of cone density calculated from each of the three graders cone identifications. S = superior, N = nasal.

**Figure 4.**
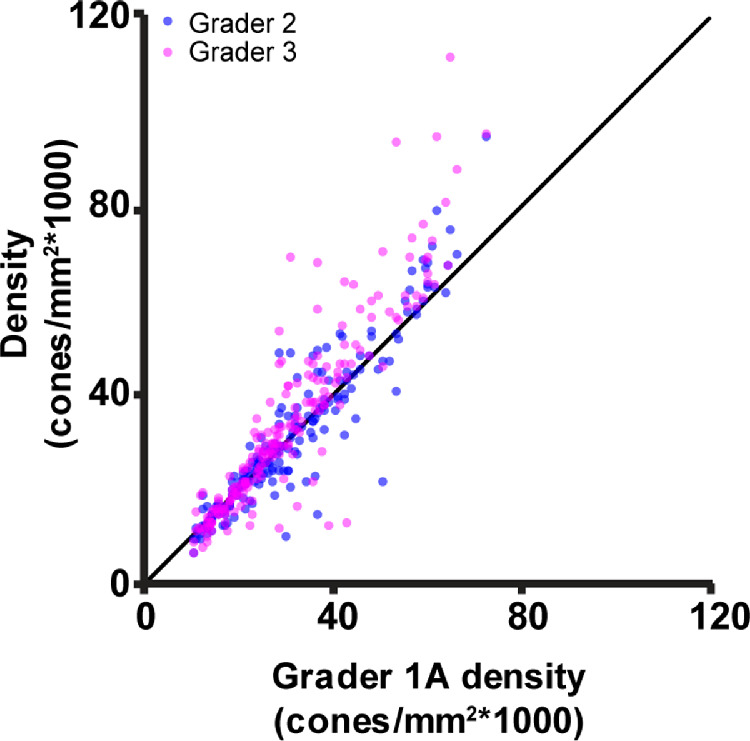
Cone density measured from graders 2 and 3 versus cone density measured by grader 1A. The three graders showed excellent agreement when measuring cone density in CHM (interclass correlation coefficient = 0.862). The *black line* depicts the line of equivalence.

Intergrader agreement was also good; graders 1 to 3 showed high reliability when manually identifying cones within the 204 ROIs (Fig. 3). The Dice coefficient was higher between graders 1A and 2 than between graders 1A and 3, or graders 2 and 3, *P* < 0.0001 for both (see Table 2). Cone density (Table 3) was not significantly different between grader 1A and grader 2 (*P* = 0.167), but identifications made by grader 3 did result in a higher cone density than grader 1A and grader 2 (*P* < 0.0001 for both). Cone density was not significantly different between grader 3 and grader 1B (*P* = 0.69). Using the scale described by Cicchetti,^41^ intergrader agreement for cone density was excellent (ICC = 0.862, CI = 0.831–0.890; Fig. 4).

**Table 3.** Mean Cone Density for Each Grader and CNN Training

Observer	Mean (SD) Cone Density cones/mm^2^
Grader 1A	30,600 (14,200)
Grader 1B	34,000 (16,100)
Grader 2	31,200 (16,300)
Grader 3	34,300 (19,800)
Normal-CNN	18,000 (6,200)
CHM-1A-CNN	30,200 (9,800)
CHM-1B-CNN	33,300 (11,400)
CHM-2-CNN	30,600 (10,700)
CHM-3-CNN	32,900 (9,900)

**Figure 5.**
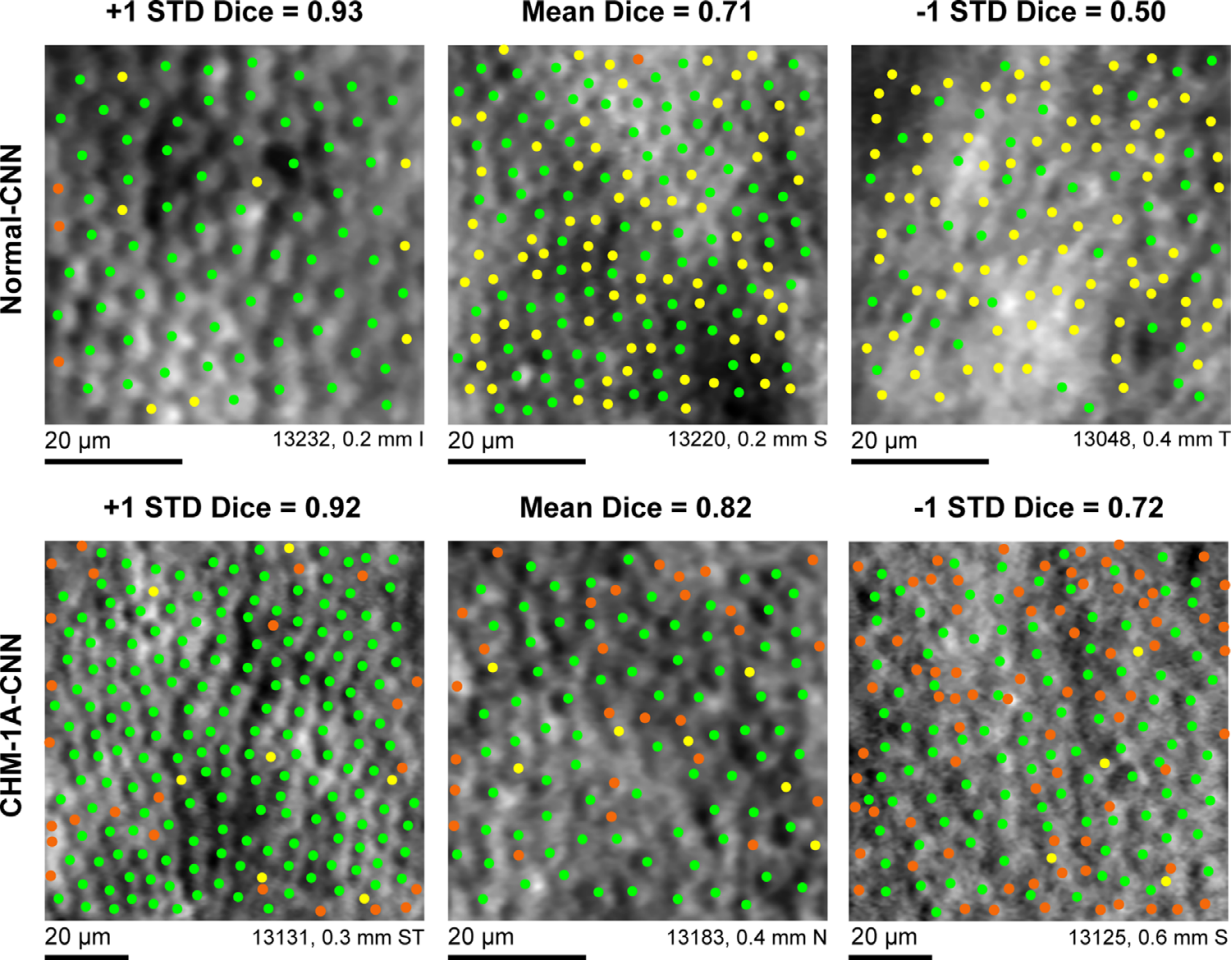
Automated cone identifications from CNN in comparison to manual cone selections by an experienced grader. *Green dots* show cones that were identified by both the CNN and by grader 1A. *Yellow dots* show cone locations selected by grader 1A but not the CNN. *Orange dots* show cone locations that were selected by the CNN but not by grader 1A. *Top row*: normal-CNN; *bottom row*: CHM-1A-CNN. The three images shown are the images corresponding to the mean and the mean ± 1 SD Dice coefficient for each CNN training condition. I = inferior, N = nasal, S = superior, T = temporal.

**Figure 6.**
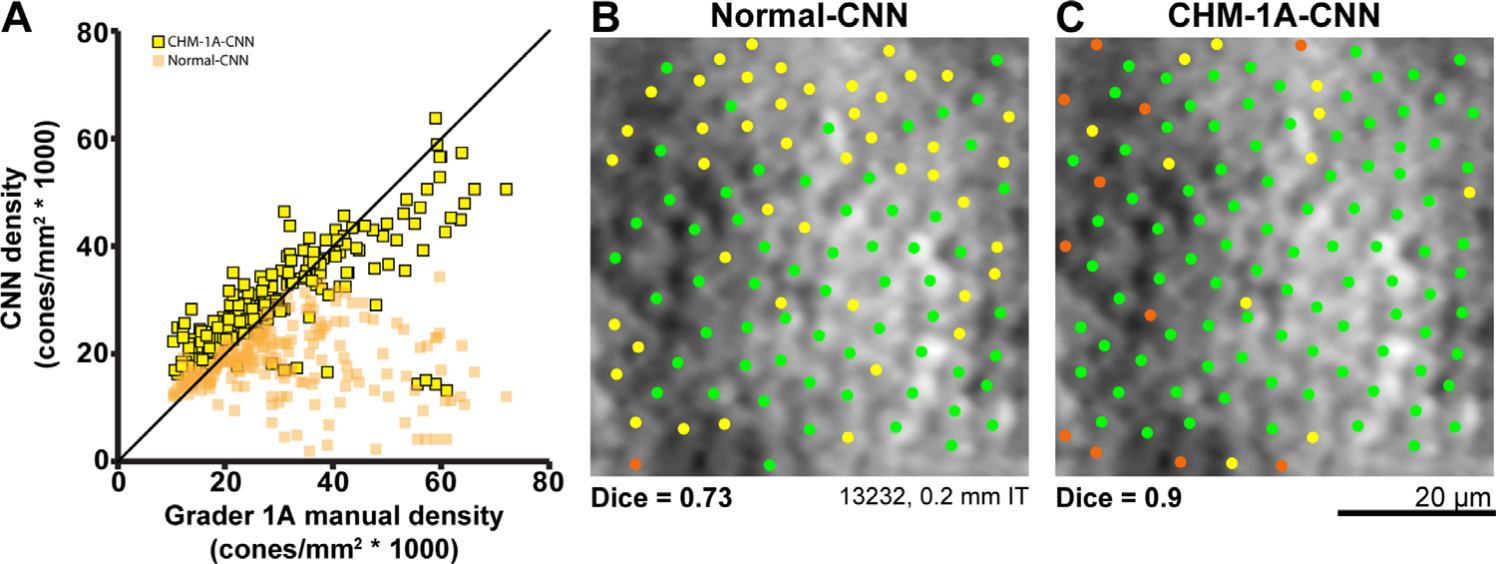
(**A**) Cone density calculations resulting from the CNN (normal-CNN, *orange squares*; CHM-1A-CNN, *yellow squares with black outline*) automated cone identifications versus the cone densities calculated from the first set of manual cone identifications made by grader 1. The normal-trained network's cone identifications resulted in lower cone densities than the manual grader. The CHM-trained network resulted in higher cone densities than the manual grader for low density images, but lower cone densities than the manual grader for high density images. The CHM-trained network always results in a higher calculated cone density than the normal-trained network. The *black line* shows the line of equivalence. (**B, C**) Automated cone identifications from the normal and CHM-trained CNNs for the same image. *Green dots* show cones that were identified by both the CNN and by grader 1A. *Yellow dots* show cone locations selected by grader 1A but not the CNN. *Orange dots* show cone locations that were selected by the CNN but not by grader 1A. **B** Normal-CNN; **C** CHM-1A-CNN. The CHM-trained CNN showed a higher number of true positives and a higher number of false positives compared to the normal-trained network. The ROI shown in the figure had the mean difference in cone density between the CHM and normal trained networks. I = inferior, T = temporal.

**Figure 7.**
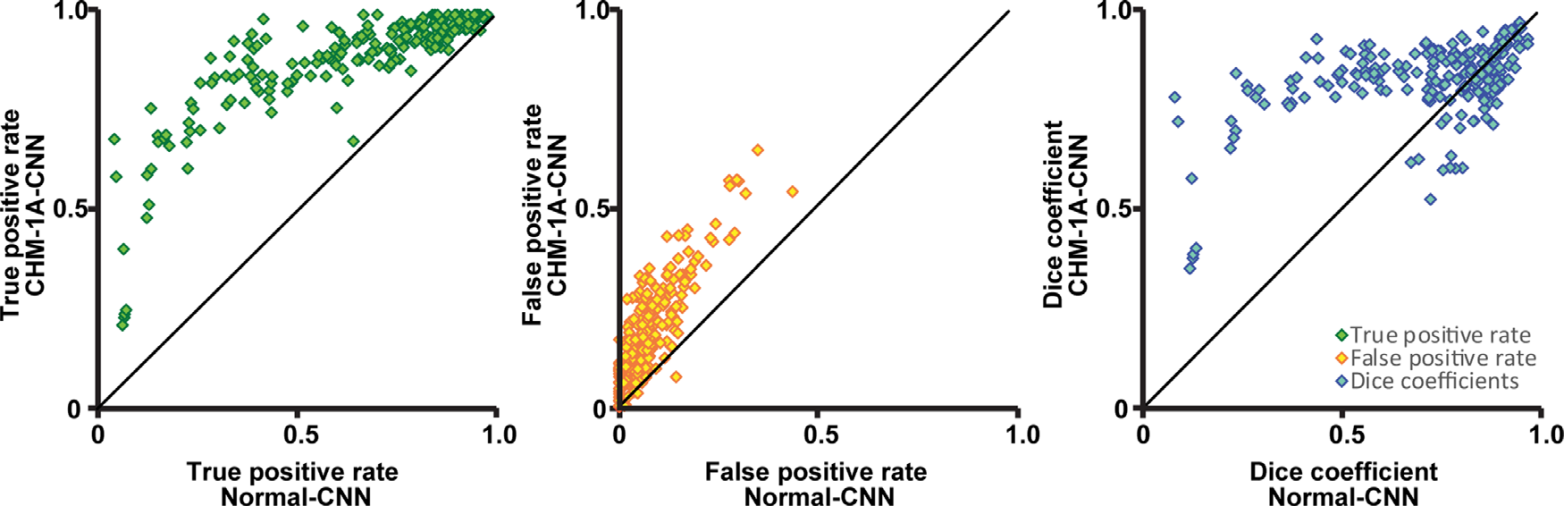
The true positive and false positive rates and Dice coefficients for CHM-1A-CNN in comparison to normal-CNN. The true positive and false positive rates for cone identifications were higher for CHM-1A-CNN in comparison to normal-CNN. Dice's coefficient showed more variability, with some images yielding a higher Dice coefficient and while others yielded a lower Dice coefficient for CHM-1A-CNN in comparison to the normal-CNN. On average, the Dice coefficient increased for CHM-1A-CNN. The *black lines* show the lines of equivalence.

**Figure 8.**
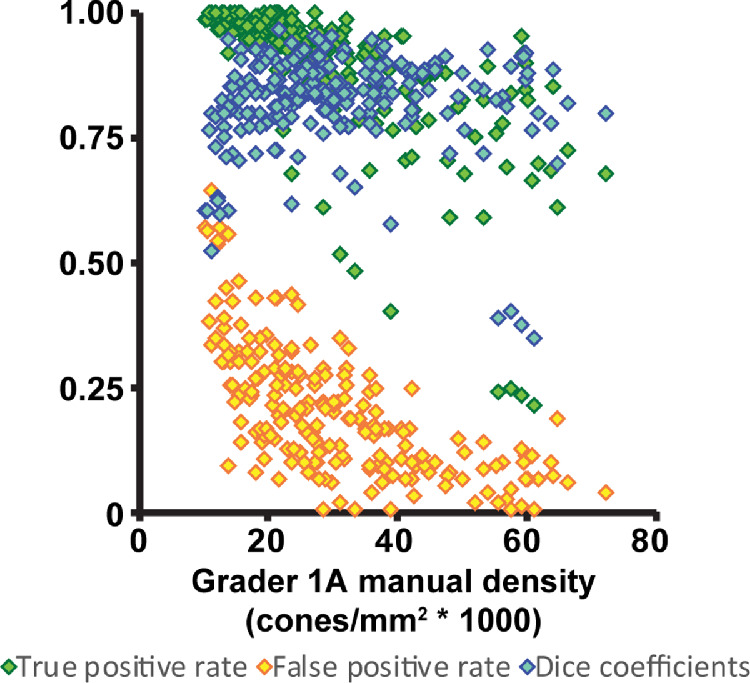
True positive and false positive rates and Dice coefficient for the CHM-1A-CNN plotted versus the calculated cone densities from grader 1A. The false positive rate is higher for ROIs that have a lower manual cone density. The true positive rate is lower for ROIs that have a higher manual cone density. The Dice coefficient depends on both the true positive and false positive rates, resulting in lower Dice coefficients for high and low manual density ROIs and higher Dice coefficients for ROIs with mid-range manual cone densities. *Green diamonds* = true positive rates; *yellow diamonds* = false positive rates; and *blue diamonds* = Dice coefficients.

The normal-CNN results showed variable success at automatically identifying cones in images of CHM (Fig. 5 top row). When using grader 1A as ground truth, the normal-CNN resulted in a Dice coefficient of 0.71 ± 0.21 (Table 4). This is lower than both the intra- and intergrader Dice measurements, *P* < 0.0001 for both. Calculated bound cone density for the normal-CNN was significantly lower than manual cone density measurements, *P* < 0.0001 (Fig. 6). Retraining the CNN on the CHM images (CHM-1A-CNN) improved automated cone identifications (see Fig. 5 bottom row). The CHM-1A-CNN yielded a higher true positive rate (0.88 ± 0.14) in comparison to the true positive rate for the normal-CNN (0.64 ± 0.26), *P* < 0.0001 (Fig. 7 and see Table 4). However, the false negative rate was also higher (*P* < 0.0001), resulting in some images showing an improved Dice coefficient, whereas others showed a reduced Dice coefficient (see Fig. 7). On average, the Dice coefficient did increase for CHM-1A-CNN in comparison to normal-CNN, *P* < 0.0001 (see Table 4). As a result of both higher true and false positive rates, CHM-1A-CNN resulted in higher cone densities for all 204 ROIs in comparison to normal-CNN (see Fig. 6). There was no statistical difference for the mean cone density measured by grader 1A and CHM-1A-CNN (*P* = 0.26). However, CHM-1A-CNN overestimated cone density in images with low manual cone density and underestimated cone density with images of high manual cone density (see Fig. 6). This resulted from an increasing false positive rate with decreasing manual cone density and a decreasing true positive rate with increasing cone density (Fig. 8).

**Table 4.** Agreement Between CNN Automatic Cone Identifications and Manual Cone Identifications

Ground Truth	Comparison CNN	True Positive Rate	False Positive Rate	Dice's Coefficient
Manual observer		Mean (SD)	Mean (SD)	Mean (SD)
Grader 1A	Normal-CNN	0.64 (0.26)	0.08 (0.07)	0.71 (0.21)
	CHM-1A-CNN	0.88 (0.14)	0.20 (0.13)	0.82 (0.10)
	CHM-1B-CNN	0.92 (0.12)	0.25 (0.14)	0.81 (0.10)
Grader 1B	CHM-1A-CNN	0.84 (0.15)	0.13 (0.11)	0.84 (0.10)
	CHM-1B-CNN	0.89 (0.13)	0.16 (0.12)	0.85 (0.09)
Grader 2	CHM-2-CNN	0.86 (0.15)	0.20 (0.17)	0.81 (0.12)
Grader 3	CHM-3-CNN	0.82 (0.15)	0.21 (0.18)	0.78 (0.11)

**Figure 9.**
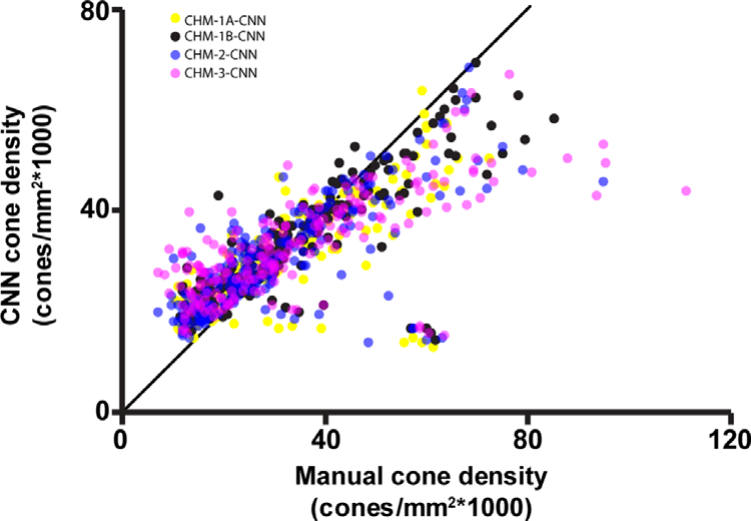
Training the CNN with images from CHM resulted in highly correlated cone densities regardless of which manual set of cone identifications was used for training. CHM-1A-CNN, CHM-1B-CNN, CHM-2-CNN, and CHM-3-CNN all overestimated cone density for ROIs with low manual densities and underestimated cone density for ROIs with high manual densities. *Yellow dots* = CHM-1A-CNN; *black dots* = CHM-1B-CNN; *blue dots* = CHM-2-CNN; and *magenta dots* = CHM-3-CNN. The *black line* shows the line of equivalence.

Similar results were found when using grader 1B, grader 2, or grader 3 to train the CNN. CHM-1B-CNN resulted in the highest Dice coefficient (*P* < .01; see Table 4). Interestingly, there was no statistical difference in the Dice coefficient when using grader 1A or grader 1B as the ground truth comparison to CHM-1B-CNN. For CHM-1A-CNN, there was a difference in Dice coefficient when using grader 1A as ground truth versus grader 1B, although this difference was small (Dice = 0.82 ± 0.10 vs. 0.81 ± 0.10, *P* < 0.001; see Table 4). There was no statistical difference in Dice coefficients between CHM-1A-CNN and CHM-2-CNN (*P* > 0.05 after correcting for multiple comparisons). CHM-3-CNN resulted in a lower Dice coefficient than CHM-1A-CNN, CHM-1B-CNN, and CHM-2-CNN (*P* < 0.001 for all). Although there were differences in the Dice coefficient, the resultant densities from CHM-1A-CNN, CHM-1B-CNN, CHM-2-CNN, and CHM-3-CNN were highly correlated (ICC = 0.944, CI = 0.931–0.956). Regardless of which graders’ manual identifications were used for training, the CHM-trained networks consistently overestimated cone density in ROIs with low manual cone density and underestimated cone density in ROIs with high manual cone density (Fig. 9).

## Discussion

Understanding the agreement between graders is important for understanding the confidence with which cone density measurements are reported in cross-sectional studies and for helping to determine limitations for being able to measure true changes in cone density over time, for example, a reduction in cone density caused by disease progression. Previous studies have shown that manual intergrader agreement for cone density measurements was excellent when experienced graders manually or semimanually identified cones in confocal images of the parafoveal normal retina.^31^^,^^42^ Excellent agreement in cone density has also been demonstrated with expert observers manually identifying cones in perifoveal normal images acquired in both the confocal and split-detection AOSLO imaging modalities.^31^ This same study found eccentricity and imaging modality had an effect on intergrader agreement, with the highest agreement coming from parafoveal confocal images and with nonconfocal split-detection yielding higher agreement than confocal images for perifoveal locations.^31^ Disease also seems to play a variable role in intergrader agreement, likely because pathological changes can cause difficulties in determining what is a cone and images from patients can have a lower image quality than those from controls. For example, a previous study found images from Stargardt disease yielded a higher manual intergrader agreement than images from retinitis pigmentosa GTPase regulator (RPGR)-associated retinopathy.^43^ Images of achromatopsia have been shown to have low repeatability and reliability for manual cone identifications.^44^ In the present study, we showed high intra- and intergrader agreement in cone density measurements in images of CHM.

We also found that manual intragrader agreement was slightly higher than manual inter-grader agreement. This is to be expected; because manual cone identifications are subjectively determined, each grader undertakes cone identification tasks using their own criteria and biases for selecting cone locations. Repeated cone selections by grader 1 separated by a minimum of 6 months showed statistically significant higher agreement in individual cone identifications than when comparing cone identifications among graders. However, this difference was small, showing that despite the subjective nature of manual cone identifications, experienced graders showed consistency and agreement in their independent, subjective evaluations of cone locations. Using the identified cone locations to calculate bound cone density showed density measurements were similar between graders; bound cone density was not statistically different between grader 1A and grader 2, although grader 3 calculated densities were slightly higher than both. However, grader 1B densities were not statistically different from grader 3, although they were higher than both grader 1A and grader 2 (see Table 3, Fig. 4). Taken all together, we believe this to show that cone density measurements between the graders are similar even if statistically significant small differences are present, that each grader's measurements in the current study are equally valid, and that cone density can be reliably measured in images of CHM.

Although repeatable and reliable, manual cone identifications are tedious to undertake. As a result, there is high interest in developing automated methods to replace the manual task of identifying cones. CNNs have shown promise for automatically identifying cones in AOSLO images of normal retina^27^ and images from patients with achromatopsia^28^ and Stargardts.^29^ In the present study, we used the open source split-detection CNN developed by Cunefare et al.^27^ and showed that the Dice coefficient for the CHM-trained split-detection CNN selections in comparison to manual selections ranged from 0.78 to 0.85 for different graders (see Table 4). This is comparable to results using the same split-detection CNN retrained for achromatopsia (Dice coefficient, 0.867)^28^ and for Stargardt's (Dice coefficient, 0.8797).^29^ As with the achromatopsia and Stargardt's studies, our study found the CNN performed better once it was trained on images from CHM in comparison to using the CNN pretrained on normal images (see Figs. 5^6^–7). This again is unsurprising; CNN's are dependent on the learning they have received through the training process so it is reasonable to expect a CNN will perform better on tasks for which it is explicitly trained. However, it is unclear whether training on disease phenotype was the sole cause of the observed improvement. There were other differences between our CHM images and the normative images used for training the CNN other than the presence or absence of disease. The split-detection training set from normal controls included images 0.5 to 2.8 mm from fixation,^27^ whereas our CHM training imaging ranged from 0.14 to 2.2 mm, with 95% of the CHM images located within 1.0 mm of fixation. As a result, the CHM training data showed higher cone densities than the normal-trained data. Thus, the improvement in the CHM-trained network may arise in part from the inclusion of additional retinal eccentricities and the higher cone densities measured in the training data in addition to disease state.

A CNN can only be expected to be as good as its training data. As a result, we would not expect the output of the CNN to show perfect agreement with manual results because manual results are not perfectly repeatable. The fact that the CNN yielded results that behaved similarly despite being trained using different graders’ cone identifications (see Fig. 9) suggests that the CNN generally learned the same principles for identifying cones in CHM regardless of which grader provided the training data. However, CNN automated cone identifications yielded lower agreement than the measured manual intra- and intergrader agreement, and it resulted in cone densities that were different from the manual graders’ densities (see Fig. 9). As a result, the present study shows that manual cone identifications remain superior to the currently available open-source CNN for automated cone selections in CHM.

Our goal in the present study was not to develop a CNN for cone identification in CHM, but rather to test the applicability of an open-source CNN to automatically identify cones in CHM. The distinction is subtle but important; our results show that the currently available open-source CNN does not have the same accuracy for identifying cones in CHM that manual graders do, but this does not mean that one cannot be developed. Indeed, we would expect that the open-source CNN would improve with additional training resources, irrespective of CHM. Previous studies have shown CNNs can improve when using multimodal information, such as paired confocal and split-detection images of the same location.^28^ In addition, CNNs trained to distinguish rods from cones are expected to perform better on images containing both photoreceptor types.^45^ The open-source CNN to which we had access for the present study was limited to using a single AOSLO imaging modality at a time and was limited to identifying cone photoreceptors rather than distinguishing rods from cones.^27^ In addition and as already mentioned, the present study included images from a different range of retinal eccentricities than the CNN training data. Retinal topography is expected to change rapidly over the central macula particularly with regard to the presence and density of rods relative to cones.^46^ Inspection of the images and CNN automated identifications post hoc showed numerous examples where the network selected rods as cones in ROIs with low manual cone density. Thus, we would expect a CNN trained with multimodal images and capable of distinguishing rods from cones would improve the agreement between the automated cone identifications and the manual graders. In addition, we expect a CNN trained with knowledge of retinal eccentricity may also improve the automated results. Including more images in the training set may also improve CNN performance. Finally, other deep learning approaches, such as a multidimensional recurrent neural network, could be explored in future studies. These efforts are worth pursuing as the advantages of accurate automated cone selections greatly surpass the requirements of manual grading. In addition to reducing the time and operator effort required to obtain manual measurements, CNNs have the potential to remove the subjective biases of manual selections and provide consistent, objective results across images and subjects.

In summary, identifying cones and measuring cone density in CHM is repeatable and reliable for manual graders. CNNs hold promise for accurate cone selections, although for CHM, and likely many other retinal diseases, CNNs will need additional improvements before their accuracy can equal or surpass manual agreement. This information will be useful as investigators commence longitudinal studies of disease progression and treatment intervention in CHM.

## References

[1] Roorda A, Romero-Borja F, Donnelly WJ III, Queener H, Hebert TJ, Campbell MCW Adaptive optics scanning laser ophthalmoscopy. *Opt Express*. 2002; 10: 405–412. 19436374 10.1364/oe.10.000405

[2] Morgan JI. The fundus photo has met its match: optical coherence tomography and adaptive optics ophthalmoscopy are here to stay. *Ophthalmic Physiol Opt*. 2016; 36: 218–239. 27112222 10.1111/opo.12289 PMC4963017

[3] Roorda A, Duncan JL Adaptive optics ophthalmoscopy. *Annu Rev Vis Sci*. 2015; 1: 19–50. 26973867 10.1146/annurev-vision-082114-035357 PMC4786023

[4] Duncan JL, Zhang Y, Gandhi J, et al. High-resolution imaging with adaptive optics in patients with inherited retinal degeneration. *Invest Ophthalmol Vis Sci*. 2007; 48: 3283–3291. 17591900 10.1167/iovs.06-1422

[5] Makiyama Y, Ooto S, Hangai M, et al. Macular cone abnormalities in retinitis pigmentosa with preserved central vision using adaptive optics scanning laser ophthalmoscopy. *PLoS One*. 2013; 8: e79447. 24260224 10.1371/journal.pone.0079447 PMC3834127

[6] Sun LW, Johnson RD, Langlo CS, et al. Assessing photoreceptor structure in retinitis pigmentosa and Usher syndrome. *Invest Ophthalmol Vis Sci*. 2016; 57: 2428–2442. 27145477 10.1167/iovs.15-18246 PMC5089122

[7] Nakatake S, Murakami Y, Funatsu J, et al. Early detection of cone photoreceptor cell loss in retinitis pigmentosa using adaptive optics scanning laser ophthalmoscopy. *Graefes Arch Clin Exp Ophthalmol*. 2019; 257: 1169–1181. 30937533 10.1007/s00417-019-04307-0

[8] Chen Y, Ratnam K, Sundquist SM, et al. Cone photoreceptor abnormalities correlate with vision loss in patients with Stargardt disease. *Invest Ophthalmol Vis Sci*. 2011; 52: 3281–3292. 21296825 10.1167/iovs.10-6538 PMC3109028

[9] Razeen MM, Cooper RF, Langlo CS, et al. Correlating photoreceptor mosaic structure to clinical findings in Stargardt disease. *Transl Vis Sci Technol*. 2016; 5: 6. 10.1167/tvst.5.2.6 PMC4790429 26981328

[10] Song H, Rossi EA, Latchney L, et al. Cone and rod loss in Stargardt disease revealed by adaptive optics scanning light ophthalmoscopy. *JAMA Ophthalmol*. 2015; 133: 1198–1203. 26247787 10.1001/jamaophthalmol.2015.2443 PMC4600048

[11] Genead MA, Fishman GA, Rha J, et al. Photoreceptor structure and function in patients with congenital achromatopsia. *Invest Ophthalmol Vis Sci*. 2011; 52: 7298–7308. 21778272 10.1167/iovs.11-7762 PMC3183969

[12] Dubis AM, Cooper RF, Aboshiha J, et al. Genotype-dependent variability in residual cone structure in achromatopsia: toward developing metrics for assessing cone health. *Invest Ophthalmol Vis Sci*. 2014; 55: 7303–7311. 25277229 10.1167/iovs.14-14225 PMC4235328

[13] Langlo CS, Patterson EJ, Higgins BP, et al. Residual foveal cone structure in CNGB3-associated achromatopsia. *Invest Ophthalmol Vis Sci*. 2016; 57: 3984–3995. 27479814 10.1167/iovs.16-19313 PMC4978151

[14] Georgiou M, Litts KM, Kalitzeos A, et al. Adaptive optics retinal imaging in CNGA3-associated achromatopsia: retinal characterization, interocular symmetry, and intrafamilial variability. *Invest Ophthalmol Vis Sci*. 2019; 60: 383–396. 30682209 10.1167/iovs.18-25880 PMC6354941

[15] Morgan JI, Han G, Klinman E, et al. High-resolution adaptive optics retinal imaging of cellular structure in choroideremia. *Invest Ophthalmol Vis Sci*. 2014; 55: 6381–6397. 25190651 10.1167/iovs.13-13454 PMC4193760

[16] Sun LW, Johnson RD, Williams V, et al. Multimodal imaging of photoreceptor structure in choroideremia. *PLoS One*. 2016; 11: e0167526. 27936069 10.1371/journal.pone.0167526 PMC5147929

[17] Syed R, Sundquist SM, Ratnam K, et al. High-resolution images of retinal structure in patients with choroideremia. *Invest Ophthalmol Vis Sci*. 2013; 54: 950–961. 23299470 10.1167/iovs.12-10707 PMC3564452

[18] Jackson K, Vergilio GK, Cooper RF, Ying GS, Morgan JIW A 2-year longitudinal study of normal cone photoreceptor density. *Invest Ophthalmol Vis Sci*. 2019; 60: 1420–1430. 30943290 10.1167/iovs.18-25904 PMC6736277

[19] Langlo CS, Erker LR, Parker M, et al. Repeatability and longitudinal assessment of foveal cone structure in Cngb3-associated achromatopsia. *Retina*. 2017; 37: 1956–1966. 28145975 10.1097/IAE.0000000000001434 PMC5537050

[20] Talcott KE, Ratnam K, Sundquist SM, et al. Longitudinal study of cone photoreceptors during retinal degeneration and in response to ciliary neurotrophic factor treatment. *Invest Ophthalmol Vis Sci*. 2011; 52: 2219–2226. 21087953 10.1167/iovs.10-6479 PMC3080173

[21] MacDonald IM, Russell L, Chan CC Choroideremia: new findings from ocular pathology and review of recent literature. *Surv Ophthalmol*. 2009; 54: 401–407. 19422966 10.1016/j.survophthal.2009.02.008 PMC2679958

[22] Coussa RG, Traboulsi EI Choroideremia: a review of general findings and pathogenesis. *Ophthalmic Genet*. 2012; 33: 57–65. 22017263 10.3109/13816810.2011.620056

[23] Aleman TS, Han G, Serrano LW, et al. Natural history of the central structural abnormalities in choroideremia: a prospective cross-sectional study. *Ophthalmology*. 2017; 124: 359–373. 27986385 10.1016/j.ophtha.2016.10.022 PMC5319901

[24] Jacobson SG, Cideciyan AV, Sumaroka A, et al. Remodeling of the human retina in choroideremia: rab escort protein 1 (REP-1) mutations. *Invest Ophthalmol Vis Sci*. 2006; 47: 4113–4120. 16936131 10.1167/iovs.06-0424

[25] Tuten WS, Vergilio GK, Young GJ, et al. Visual function at the atrophic border in choroideremia assessed with adaptive optics microperimetry. *Ophthalmol Retina*. 2019; 3: 888–899. 31235310 10.1016/j.oret.2019.05.002 PMC6778501

[26] Morgan JIW Adaptive optics retinal imaging techniques and clinical applications. In: Guenther R Steel D (eds), *Encyclopedia of Modern Optics*, 2nd edition Oxford: Elsevier; 2018: 72–84.

[27] Cunefare D, Fang L, Cooper RF, Dubra A, Carroll J, Farsiu S Open source software for automatic detection of cone photoreceptors in adaptive optics ophthalmoscopy using convolutional neural networks. *Sci Rep*. 2017; 7: 6620. 28747737 10.1038/s41598-017-07103-0 PMC5529414

[28] Cunefare D, Langlo CS, Patterson EJ, et al. Deep learning based detection of cone photoreceptors with multimodal adaptive optics scanning light ophthalmoscope images of achromatopsia. *Biomed Opt Express*. 2018; 9: 3740–3756. 30338152 10.1364/BOE.9.003740 PMC6191607

[29] Davidson B, Kalitzeos A, Carroll J, et al. Automatic cone photoreceptor localisation in healthy and Stargardt afflicted retinas using deep learning. *Sci Rep*. 2018; 8: 7911. 29784939 10.1038/s41598-018-26350-3 PMC5962538

[30] Bennett AG, Rudnicka AR, Edgar DF Improvements on Littmann's method of determining the size of retinal features by fundus photography. *Graefes Arch Clin Exp Ophthalmol*. 1994; 232: 361–367. 8082844 10.1007/BF00175988

[31] Morgan JIW, Vergilio GK, Hsu J, Dubra A, Cooper RF The reliability of cone density measurements in the presence of rods. *Transl Vis Sci Technol*. 2018; 7: 21. 10.1167/tvst.7.3.21 PMC6016505 29946495

[32] Dubra A, Sulai Y Reflective afocal broadband adaptive optics scanning ophthalmoscope. *Biomed Opt Express*. 2011; 2: 1757–1768. 21698035 10.1364/BOE.2.001757 PMC3114240

[33] Scoles D, Sulai YN, Langlo CS, et al. In vivo imaging of human cone photoreceptor inner segments. *Invest Ophthalmol Vis Sci*. 2014; 55: 4244–4251. 24906859 10.1167/iovs.14-14542 PMC4095721

[34] Salmon AE, Cooper RF, Langlo CS, Baghaie A, Dubra A, Carroll J An automated reference frame selection (ARFS) algorithm for cone imaging with adaptive optics scanning light ophthalmoscopy. *Transl Vis Sci Technol*. 2017; 6: 9. 10.1167/tvst.6.2.9 PMC5381332 28392976

[35] Dubra A, Harvey Z. Registration of 2D images from fast scanning ophthalmic instruments. *Biomedical Image Registration: 4th International Workshop, WBIR 2010, Lübeck, Germany, July 11–13, 2010. Proceedings*. 2010; 6204: 60–71. 10.1007/978-3-642-14366-3_6 PMC13282932 42328045

[36] Bedggood P, Metha A. De-warping of images and improved eye tracking for the scanning laser ophthalmoscope. *PLoS One*. 2017; 12: e0174617. 28369065 10.1371/journal.pone.0174617 PMC5378343

[37] Chen M, Cooper RF, Han GK, Gee J, Brainard DH, Morgan JI Multi-modal automatic montaging of adaptive optics retinal images. *Biomed Opt Express*. 2016; 7: 4899–4918. 28018714 10.1364/BOE.7.004899 PMC5175540

[38] Dice LR. Measures of the amount of ecologic association between species. *Ecology*. 1945; 26: 297–302.

[39] Cooper RF, Wilk MA, Tarima S, Carroll J Evaluating descriptive metrics of the human cone mosaic. *Invest Ophthalmol Vis Sci*. 2016; 57: 2992–3001. 27273598 10.1167/iovs.16-19072 PMC4898203

[40] Bland JM, Altman DG. Statistical methods for assessing agreement between two methods of clinical measurement. *Lancet*. 1986; 1: 307–310. 2868172

[41] Cicchetti DV Guidelines, criteria, and rules of thumb for evaluating normed and standardized assessment instruments in psychology. *Psychol Assess*. 1994; 6: 284–290.

[42] Garrioch R, Langlo C, Dubis AM, Cooper RF, Dubra A, Carroll J Repeatability of in vivo parafoveal cone density and spacing measurements. *Optom Vis Sci*. 2012; 89: 632–643. 22504330 10.1097/OPX.0b013e3182540562 PMC3348369

[43] Tanna P, Kasilian M, Strauss R, et al. Reliability and repeatability of cone density measurements in patients with Stargardt disease and RPGR-associated retinopathy. *Invest Ophthalmol Vis Sci*. 2017; 58: 3608–3615. 28738413 10.1167/iovs.17-21904 PMC5525557

[44] Abozaid MA, Langlo CS, Dubis AM, Michaelides M, Tarima S, Carroll J Reliability and repeatability of cone density measurements in patients with congenital achromatopsia. *Adv Exp Med Biol*. 2016; 854: 277–283. 26427422 10.1007/978-3-319-17121-0_37 PMC4839591

[45] Cunefare D, Huckenpahler AL, Patterson EJ, Dubra A, Carroll J, Farsiu S RAC-CNN: multimodal deep learning based automatic detection and classification of rod and cone photoreceptors in adaptive optics scanning light ophthalmoscope images. *Biomed Opt Express*. 2019; 10: 3815–3832. 31452977 10.1364/BOE.10.003815 PMC6701534

[46] Curcio CA, Sloan KR, Kalina RE, Hendrickson AE Human photoreceptor topography. *J Comp Neurol*. 1990; 292: 497–523. 2324310 10.1002/cne.902920402

